# Fatal Case of COVID-19-Associated Acute Hemorrhagic Leukoencephalitis Without Respiratory Involvement: A Diagnostic and Therapeutic Challenge

**DOI:** 10.7759/cureus.89900

**Published:** 2025-08-12

**Authors:** Benjamin Easow, Saikiran Mandyam, John Vallas, Kenneth Hau, Fnu Anshul

**Affiliations:** 1 Internal Medicine, Southeast Health Medical Center, Dothan, USA; 2 Critical Care Medicine, Southeast Health Medical Center, Dothan, USA

**Keywords:** acute hemorrhagic leukoencephalitis ahle, covid-19, general internal medicine, infectious disease medicine, intravenous immunoglobulins (ivig)

## Abstract

We report a rare and fatal case of acute hemorrhagic leukoencephalitis (AHLE) as the sole manifestation of COVID-19 in a 76-year-old woman who presented without respiratory symptoms. The patient was admitted with an acute altered mental status following a recent fall and a positive SARS-CoV-2 test. Initial imaging showed a small subdural hematoma; however, further evaluation revealed widespread white matter changes and petechial hemorrhages on MRI, consistent with AHLE. Despite aggressive treatment, including intravenous immunoglobulin (IVIG) and high-dose corticosteroids, the patient showed minimal neurological recovery. Cerebrospinal fluid (CSF) studies revealed elevated protein with no infectious or autoimmune markers, and the brain biopsy was non-contributory. She was ultimately transitioned to hospice care. This case underscores the importance of considering AHLE in COVID-19 patients presenting with isolated neurological deterioration, even in the absence of pulmonary involvement. Prompt recognition and escalation of care are crucial, although the prognosis remains poor.

## Introduction

Acute hemorrhagic leukoencephalitis (AHLE), also known as Weston-Hurst disease, is a rare and fulminant variant of acute disseminated encephalomyelitis (ADEM). Both are post-infectious demyelinating syndromes that can present with rapid neurological decline, often making early differentiation difficult [1,2]. AHLE is characterized by diffuse cerebral inflammation, demyelination, and hemorrhage, and carries a significantly worse prognosis than ADEM [3].

The emergence of COVID-19 has broadened the recognized spectrum of neurological complications associated with viral infections, including rare instances of AHLE [4-6]. While most COVID-19-related neurological syndromes occur concurrently with or following respiratory symptoms, the case we present is notable for the complete absence of pulmonary involvement.

We describe the case of a 76-year-old woman who developed AHLE as the sole manifestation of COVID-19, presenting exclusively with neurological symptoms. This case highlights the diagnostic complexity in distinguishing AHLE from mimics such as Guillain-Barré syndrome (GBS), ADEM, acute necrotizing encephalopathy (ANE), and the Marburg variant of multiple sclerosis [3,7]. It also underscores the importance of prompt neuroimaging, cerebrospinal fluid (CSF) analysis, and early escalation to immunotherapy in patients with rapidly progressive neurological deterioration [8].

## Case presentation

A 76-year-old woman with a history of hypertension, hyperlipidemia, chronic kidney disease stage IIIa, prior transient ischemic attack, colon cancer status post-resection, Meniere’s disease with a right cochlear implant, and prior anterior cervical fusion (C4-C6) presented to the emergency department with acute altered mental status. According to her family, she had sustained a fall two to three weeks prior and appeared unusually fatigued the day before admission. She was incidentally diagnosed with COVID-19 at an outside facility, despite having no respiratory symptoms.

On arrival, she was febrile, tachycardic, and tachypneic, with a Glasgow Coma Scale (GCS) score of 10 (E4, V2, M4). Emergency medical services noted left-sided anisocoria, prompting a code stroke activation. On examination, she was confused and exhibited right-sided weakness, global areflexia, and soft tissue bruising.

**Figure 1:**
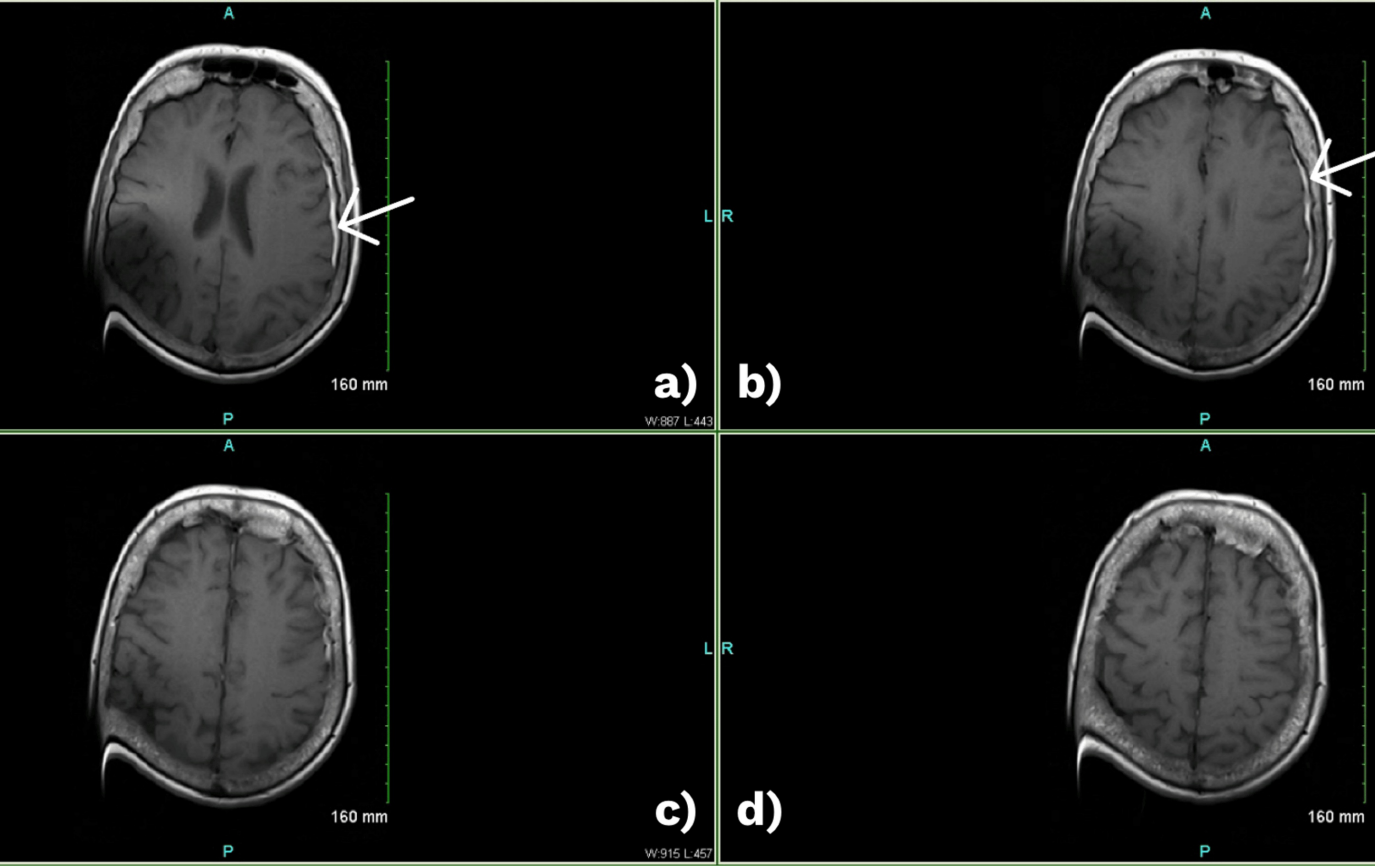
Initial MRI brain (axial T1-weighted image) a, b: subtle crescentic extra-axial hypointensity (white arrows) along the left temporoparietal convexity consistent with a small subdural hematoma (~3.1 mm); c, d: no acute infarction, mass effect, or midline shift. Image quality is limited by susceptibility artifact from the right cochlear implant.

Initial laboratory evaluation revealed a normal white blood cell count, low bicarbonate (19 mmol/L), and mild respiratory alkalosis (Tables 1, 2). A non-contrast CT of the head demonstrated a small left temporoparietal subdural hematoma (3.1 mm) without midline shift. CT angiography and perfusion imaging ruled out large vessel occlusion but showed moderate-to-high-grade vertebral artery stenosis and possible cervical hardware loosening. MRI of the brain was limited by cochlear implant artifact but showed scattered T2 hyperintensities in the white matter (Figure 1). Lumbar puncture revealed albuminocytologic dissociation (CSF protein 118 mg/dL; 0 white blood cells/μL) (Table 3).

**Table 1:** General laboratory investigations on admission PCR: polymerase chain reaction

Parameter	Patient Value	Reference Range
White Blood Cell Count	7.8 x10^3/μL	4.0–11.0 x10^3/μL
Hemoglobin	15.3 g/dL	12.0–16.0 g/dL (female)
Platelet Count	158 x10^3/μL	150–450 x10^3/μL
Sodium	136 mmol/L	135–145 mmol/L
Potassium	4.1 mmol/L	3.5–5.1 mmol/L
Bicarbonate	19 mmol/L	22–29 mmol/L
Creatinine	1.24 mg/dL	0.6–1.3 mg/dL
Calcium	10.3 mg/dL	8.5–10.5 mg/dL
Creatine Kinase (CK)	23 U/L	30–200 U/L
Ammonia	23 µmol/L	11–35 µmol/L
COVID-19 PCR	Positive	Negative
Urinalysis and Culture	Negative	Negative
Lactate	Negative	<2.0 mmol/L
Sputum Culture	Normal flora	No pathogenic growth

**Table 2:** Arterial blood gas (ABG) analysis pH: potential of hydrogen; pCO₂: partial pressure of carbon dioxide; pO₂: partial pressure of oxygen; HCO₃⁻: bicarbonate

Parameter	Patient Value	Reference Range
pH	7.402	7.35–7.45
pCO₂	28.5 mmHg	35–45 mmHg
pO₂	491.9 mmHg	75–100 mmHg (on room air)
HCO₃⁻	17.7 mmol/L	22–29 mmol/L

**Table 3:** Cerebrospinal fluid (CSF) studies VDRL: venereal disease research laboratory; PCR: polymerase chain reaction

Parameter	Patient Value	Reference Range
CSF Protein	118 mg/dL	15–45 mg/dL
CSF Glucose	58 mg/dL	40–70 mg/dL
CSF White Blood Cells	0 /μL	0–5 /μL
CSF Red Blood Cells	31 /μL	0 /μL
CSF Appearance	Clear	Clear
Meningitis/Encephalitis PCR Panel	Negative	Negative
CSF West Nile IgM	<0.90	<0.90 (Negative)
CSF VDRL (Syphilis)	Negative	Nonreactive

**Figure 2:**
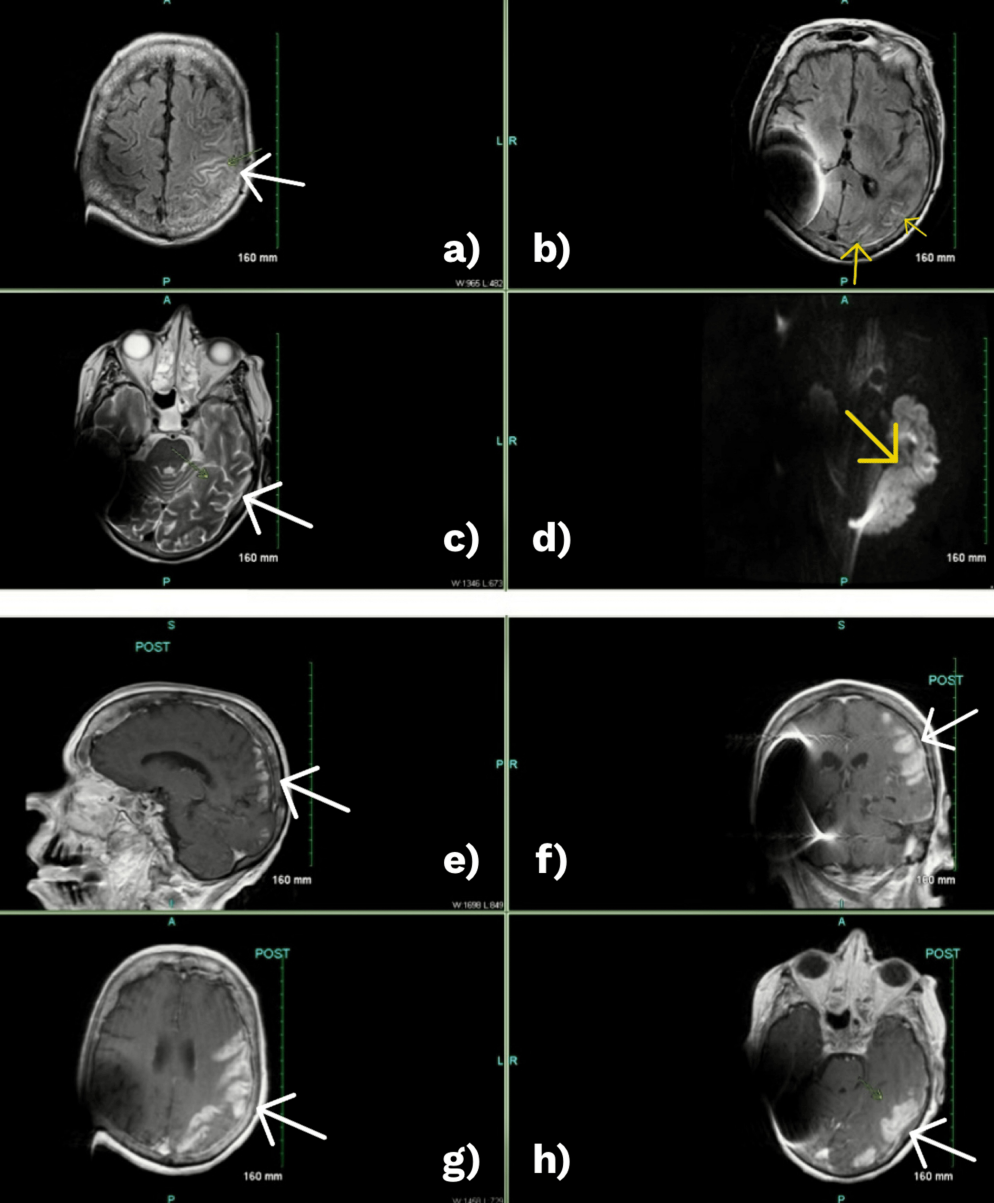
Follow-up MRI brain on hospital day 12 MRI of the brain demonstrating evolution of findings consistent with acute hemorrhagic leukoencephalitis (AHLE). a: axial fluid attenuated inversion recovery (FLAIR) image showing cortical hyperintensity and sulcal effacement in the left parietal region (white arrow); b: axial T1-weighted image revealing petechial hemorrhages and mass effect with early midline shift (yellow arrows); c: axial T2-weighted image highlighting vasogenic edema in the left cerebellar hemisphere (white arrow); d: diffusion-weighted image (DWI) showing restricted diffusion in the left hemisphere (yellow arrow); e–g: post-contrast T1-weighted sagittal, coronal, and axial images demonstrating cortical and leptomeningeal enhancement with associated mass effect and hemorrhages (white arrows); h: post-contrast axial image at the level of the brainstem showing additional abnormal enhancement (white arrow).

The patient was admitted to the intensive care unit (ICU) and was later intubated due to worsening mental status and respiratory decline. On hospital day 12, repeat MRI revealed new cortical and leptomeningeal enhancement, vasogenic edema, and petechial hemorrhages in the left frontoparietal region (Figure 2), raising concern for acute hemorrhagic meningoencephalitis.

**Figure 3:**
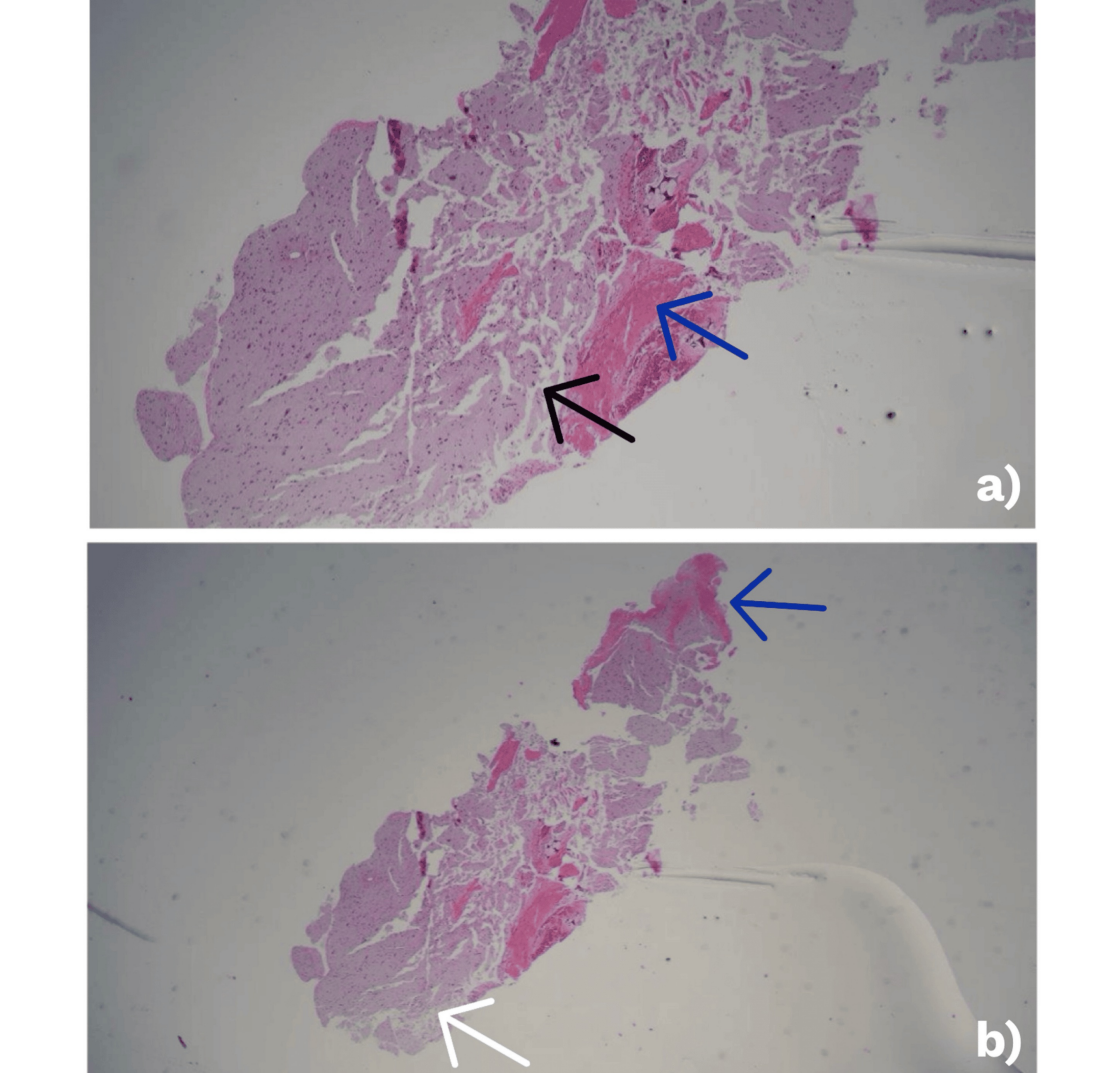
Brain biopsy histopathology (hematoxylin and eosin stain, low magnification) a: shows cortical parenchyma with scattered hemosiderin-laden macrophages (black arrow) and fibrinoid necrosis of a small vessel (blue arrow), consistent with prior microhemorrhage; b: demonstrates a necrotic vessel (blue arrow) with surrounding reactive gliosis (white arrow). No evidence of demyelination, malignancy, or infection is observed.

An extensive workup was unrevealing. CSF cultures and a comprehensive encephalitis polymerase chain reaction (PCR) panel were negative (Table 3). Serum myelin oligodendrocyte glycoprotein (MOG) and aquaporin-4 antibodies were also negative. Electroencephalography (EEG) demonstrated moderate diffuse slowing, consistent with a metabolic or toxic encephalopathy. Brain biopsy revealed gliosis and hemosiderin-laden macrophages, without evidence of malignancy, demyelination, or infection (Figure 3).

Given the encephalopathy, albuminocytologic dissociation, global areflexia, and white matter changes, GBS was initially suspected. She received intravenous immunoglobulin (IVIG) at 0.4 g/kg/day for five days, with no clinical improvement. As her condition worsened and imaging findings progressed, high-dose intravenous methylprednisolone (1 g daily for five days) was initiated for suspected AHLE. Empiric acyclovir was also started but later discontinued following negative viral studies.

During hospitalization, the patient developed *Pseudomonas aeruginosa* pneumonia, which was treated with piperacillin-tazobactam. Despite the escalation of therapy, her neurological status remained poor. She exhibited intermittent nonverbal responses and eye tracking but continued to show profound encephalopathy, right-sided weakness, and global areflexia.

Serial MRIs confirmed worsening white matter edema and petechial hemorrhages. Repeated CSF analyses consistently showed elevated protein without infectious or autoimmune markers. A second brain biopsy again demonstrated hemosiderin-laden macrophages and mild atherosclerosis, but no evidence of malignancy or demyelination.

After multidisciplinary discussion and comprehensive care, her family elected for comfort-focused measures. She was discharged to home hospice, where she passed away peacefully two weeks later.

## Discussion

AHLE, also known as Weston Hurst disease, is a fulminant demyelinating disorder characterized by widespread white matter inflammation, necrosis, and hemorrhage. It is considered a hyperacute and often fatal variant of ADEM, typically occurring after viral infections or vaccinations [3]. With the global spread of COVID-19, neurological complications such as AHLE have been increasingly recognized, although they remain rare.

While COVID-19 is primarily associated with respiratory complications, it also exerts significant effects on the central nervous system (CNS). A growing body of evidence highlights postinfectious neuroinflammatory syndromes, including encephalitis, necrotizing encephalopathy, ADEM, and AHLE [1,2,4]. In such cases, SARS-CoV-2 is believed to trigger a hyperinflammatory cascade via cytokine storm and molecular mimicry, resulting in immune-mediated demyelination and vascular injury [5].

In this case, a woman in her 70s presented with encephalopathy in the absence of respiratory symptoms and was ultimately diagnosed with AHLE. Her clinical course was marked by rapid neurological decline, albuminocytologic dissociation in the CSF, and progressive imaging findings of white matter edema and petechial hemorrhages. Despite receiving standard immunomodulatory therapy, including IVIG and high-dose corticosteroids, she exhibited no significant neurological recovery, consistent with prior reports of poor outcomes in COVID-19-associated AHLE [4,8].

Manzano et al., in a systematic review, reported that AHLE cases linked to COVID-19 commonly involve older adults, are preceded by systemic illness, often show hemorrhagic features on imaging, and carry a high mortality rate despite timely treatment [8]. This aligns with our case, where initial features resembling GBS prompted empiric IVIG therapy, but subsequent deterioration and MRI findings led to a revised diagnosis and escalation to corticosteroids.

Differentiating AHLE from other causes of fulminant CNS deterioration, such as the Marburg variant of multiple sclerosis and ANE, is essential. The Marburg variant is typically monophasic, occurs in younger individuals, and features massive demyelination with polyclonal lymphocytic infiltration [3]. ANE, by contrast, is characterized by symmetric thalamic lesions and elevated CSF protein without pleocytosis [7]. In our patient, asymmetrical lesions, sparing of the thalami, petechial hemorrhages, and rapid clinical decline favored a diagnosis of AHLE.

As of late 2021, fewer than 15 biopsy-confirmed cases of COVID-19-associated AHLE had been reported in the literature [8]. Therapies used have included high-dose corticosteroids, IVIG, plasma exchange, and experimental immunotherapies, though overall prognosis remains poor [6,8]. The lack of clinical response in our patient and the limited availability of histopathologic confirmation in most reported cases underscore the need for heightened clinical suspicion and a deeper understanding of the disease pathophysiology.

This case adds to the emerging recognition of AHLE as a rare but severe neurological complication of COVID-19, particularly in patients without respiratory involvement. It highlights the importance of early neuroimaging, timely CSF analysis, and a multidisciplinary approach to diagnosis and management. As the pandemic continues to evolve, further research is needed to clarify prognostic markers and guide optimal therapeutic strategies for COVID-19-associated AHLE.

## Conclusions

AHLE is a rare, fulminant demyelinating disorder that may occur as a severe postinfectious neurological complication of COVID-19, even in the absence of respiratory symptoms. This case highlights the importance of maintaining a high index of suspicion for AHLE in patients with rapidly progressive neurological decline and atypical imaging findings, particularly when standard infectious, autoimmune, and neoplastic workups are unrevealing. Early ICU triage, prompt neuroimaging, and timely initiation of immunomodulatory therapy are critical components of care; however, the prognosis remains poor despite intervention. This case underscores the need for further investigation into the pathophysiology of AHLE and the development of more effective therapeutic strategies for COVID-19-associated neuroinflammatory syndromes.
